# Multi-Objective Optimization of Laser Cleaning Quality of Q390 Steel Rust Layer Based on Response Surface Methodology and NSGA-II Algorithm

**DOI:** 10.3390/ma17133109

**Published:** 2024-06-25

**Authors:** Guolong Wang, Jian Deng, Jieheng Lei, Wenjie Tang, Wujiang Zhou, Zeyong Lei

**Affiliations:** 1 School of Mechanical Engineering, University of South China, Hengyang 421001, China; wgl18008453785@sina.com (G.W.); 2015002047@usc.edu.cn (J.D.); twj520803@sina.com (W.T.); z1962607764@163.com (W.Z.); 2 School of Electrical Engineering, University of South China, Hengyang 421001, China; 2016000038@usc.edu.cn

**Keywords:** laser cleaning, carbon steel, response surface methodology, NSGA-II algorithm, multi-objective optimization

## Abstract

To improve the laser cleaning surface quality of rust layers in Q390 steel, a method of determining the optimal cleaning parameters is proposed that is based on response surface methodology and the second-generation non-dominated sorting genetic algorithm (NSGA-II). It involves constructing a mathematical model of the input variables (laser power, cleaning speed, scanning speed, and repetition frequency) and the objective values (surface oxygen content, rust layer removal rate, and surface roughness). The effects of the laser cleaning process parameters on the cleaning surface quality were analyzed in our study, and accordingly, NSGA-II was used to determine the optimal process parameters. The results indicate that the optimal process parameters are as follows: a laser power of 44.99 W, cleaning speed of 174.01 mm/min, scanning speed of 3852.03 mm/s, and repetition frequency of 116 kHz. With these parameters, the surface corrosion is effectively removed, revealing a distinct metal luster and meeting the standard for surface treatment before welding.

## 1. Introduction

In the construction of nuclear power plants, the use of embedded steel rebar and steel plate components is ubiquitous, and among these components, Q390 steel plates are commonly used [1,2]. There is typically a loose rust layer on the surface, and in the welding process, water absorbed by the rust layer decomposes and produces a large amount of hydrogen, which cannot be discharged in time during the cooling process of the weld. The gases accumulate in the weld, forming pores and other defects, which significantly affects the homogeneity of the metal and the joint quality. Therefore, it is necessary to properly treat the surface of Q390 steel plates before welding. Although the commonly used treatment methods, such as manual grinding, mechanical grinding, and chemical cleaning, have practical applications, they also have limitations [3,4].

As an advanced manufacturing technology, laser cleaning has been widely used in various industrial fields, including electronics and semiconductors [5], healthcare [6], automotive manufacturing [7], aerospace [8], and heritage protection [9]. As industrial demand grows, research on the process parameters, surface quality, and performance of laser cleaning is expanding. For example, Ma et al. [10] conducted single-factor experiments to investigate the effects of laser cleaning on the rust layer of Q345 steel used in mining machinery. They specifically examined the influences of laser power and spot overlap rate on the post-cleaning surface integrity, ultimately determining optimal cleaning process parameters. Separately, Yao et al. [11] studied methods for cleaning rust layers on AH36 steel surfaces, successfully reducing surface roughness and enhancing corrosion resistance. Narayanan et al. [12] systematically observed the effects of varying parameters such as scanning speed and the number of passes on the removal depth, surface profile, roughness, and hardness of processed surfaces. In addition, Li et al. [3] explored the impact of laser scanning speed on the cleaning quality of Q345 steel, finding that the optimal cleaning effect was achieved at 3000 mm/s and noting a trend where oxygen content decreased initially and then increased as speed increased from 1000 mm/s to 6000 mm/s post-cleaning. Finally, Wu et al. [13] investigated how laser power and cleaning speed affected the macroscopic and microscopic surface morphology of cleaned specimens, revealing the formation of a re-melted layer on the substrate surface post-cleaning, which correspondingly enhanced its corrosion resistance. The types of lasers used by these researchers are shown in Table 1.

However, despite the progress made in existing research, studies in the field of laser cleaning have mainly focused on the impact of a single factor on the cleaning effect of the substrate, and few studies have considered the relationships between multiple factors and their overall impact on the quality of metal surface cleaning. In addition, genetic algorithms have not been widely used to optimize process parameters in the field of laser cleaning. The optimization strategy of response surface methodology (RSM) combined with a genetic algorithm is used in other fields such as laser cladding [14,15,16,17,18]. Therefore, in the present study, this method was introduced to the field of laser cleaning.

The rust layer of Q390 carbon steel was selected as the subject of study, and a fiber-optic pulsed laser was used to conduct experiments. Using the Box–Behnken design methodology, a mathematical model for the relationships between the laser cleaning process parameters (energy density, scanning speed, and cleaning speed) and the parameters of the surface quality assessment after cleaning (oxygen content, removal rate, and surface roughness) was developed. By analyzing the interaction patterns under different parameter combinations, this study attempted to optimize the impact indicators using a combination of response surface-based surrogate models and multi-objective genetic algorithms, thereby determining the optimal laser cleaning process parameters. This approach provides a scientific methodological basis and technical support for the laser cleaning of various carbon steels in real-life production.

## 2. Experimental Conditions and Methods

### 2.1. Materials and Equipment

**Figure 1:**
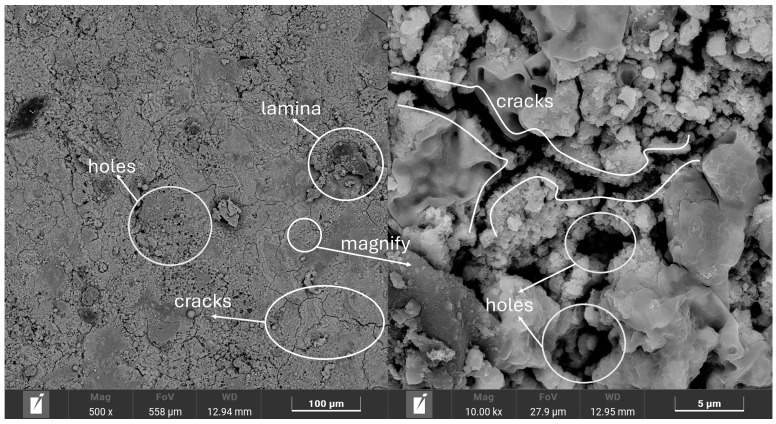
Surface morphology of the rust layer.

**Figure 2:**
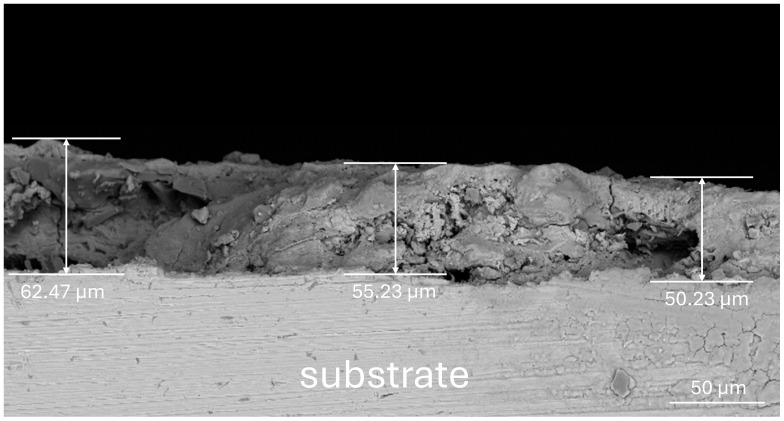
Cross-sectional morphology of the rust layer.

**Figure 3:**
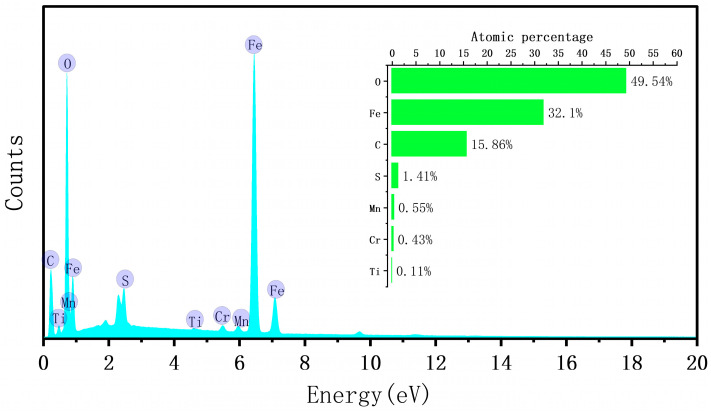
Elemental distribution of the rust layer determined via energy-dispersive X-ray spectroscopy.

In this study, Q390 steel was selected as the experimental material, and its chemical composition is presented in Table 2. The surface rust layer of the steel was formed in the natural environment, and a scanning electron microscopy (SEM) image of the sample surface (Figure 1) indicated that the rust layer was covered with a large number of cracks and pores. This indicated that the surface structure was relatively loose, making it difficult to block water and oxygen penetration; therefore, further corrosion of the interior was easily accelerated. Analysis of SEM images of the sample sections (Figure 2) revealed that the thickness of the rust layer varied between 50 and 65 μm, with significant positional differences. The cross-sectional micromorphology exhibited large pores in the middle of the rust layer and cracks in the upper locations, confirming that the structure of the rust layer was loose. The rust layer was not uniformly distributed on the specimen surface but grew from the surface to the internal pores. In addition, Figure 3 shows the results of an elemental content analysis of the surface rust layer; the rust was mainly composed of oxygen and iron, and it also contained carbon and trace amounts of other elements. The main thermophysical parameters of substrate and rust layer are shown in Table 3.

**Figure 4:**
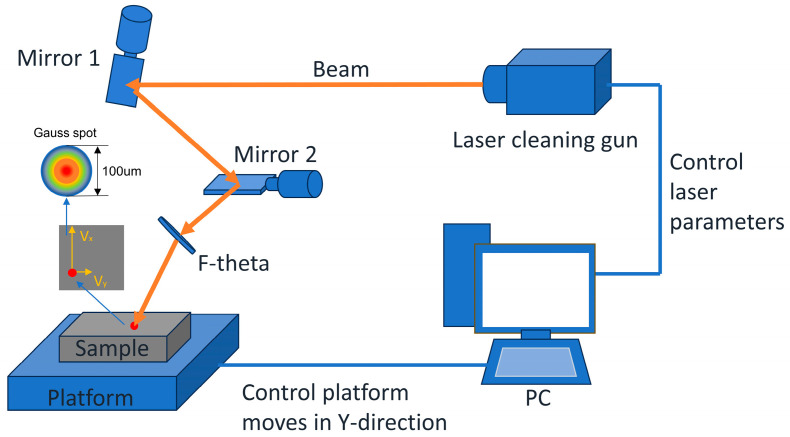
Schematic of the laser cleaning system.

In the experiment, a fiber pulsed laser (HLCM-100W, Han’S Laser, Shenzhen, China) was used as the cleaning equipment. It had various adjustable parameters, such as the laser energy density, scanning speed, and repetition frequency, which were precisely adjusted through the control system. The detailed workflow of the experiment is shown in Figure 4. In this process, the laser beam was reflected through a scanning galvanometer, and a precise laser cleaning operation was performed in the X-direction. Simultaneously, the sample was moved along the Y-direction by controlling the two-dimensional moving platform, and its speed was defined as the laser cleaning speed. The main parameter ranges of the laser cleaning platform are presented in Table 4.

### 2.2. Experimental Design

In the pre-experiment stage, the surface rust layer of the sample was unevenly distributed, which made it difficult to accurately control the process parameters of single-laser cleaning and could have easily lead to ablation of oxide residues in some areas. Therefore, the high-power and low-power double-laser cleaning method was adopted during the secondary cleaning, and differences in the laser absorption rate of the material were utilized for process refinement [20,21,22]. The parameters of the second laser cleaning process significantly affect the quality of the cleaned surface.

To better investigate the effects of the parameters of the secondary laser cleaning process on the cleaning quality, the parameters were optimized using the RSM according to the equation of regression between each of the factors and the response values. The RSM is more effective than other methods in establishing predictive models for inputs and outputs, is suitable for precise optimization of continuous variables, and offers greater flexibility in the post-experimental phase. It allows for detailed exploration of nonlinear relationships and complex interactions among variables. By inputting the independent variables of laser cleaning process parameters and describing the degrees of correlation between multiple response values and the input factors, which are measurable, controllable, and continuous for *k* independent factors, the response function can be expressed as [23]

$$y=f(x_{1},x_{2},x_{3},\ldots ,x_{k-1},x_{k}).$$

Its quadratic regression equation is [24,25,26]

$$y=b_{0}+\sum_{i=1}^{k}b_{i}x_{i}+\sum_{i=1}^{k}b_{ij}x_{ii}^{2}+\sum_{i=1}^{k}\sum_{j=1}^{k}b_{ij}x_{i}x_{j}+\varepsilon .$$

A three-factor, three-level Box–Behnken design (BBD) was used to design the experimental plan, and the RSM was applied to optimize the parameters of secondary laser cleaning in the laser cleaning process. The laser power (P), laser cleaning speed (V_x_), laser repetition frequency (f), and laser scanning speed (V_y_) were considered as independent variables for laser cleaning and rust removal. According to the results of previous experiments, the experiment levels were coded using the statistical software Design Expert 13, as shown in Table 5.

**Figure 5:**
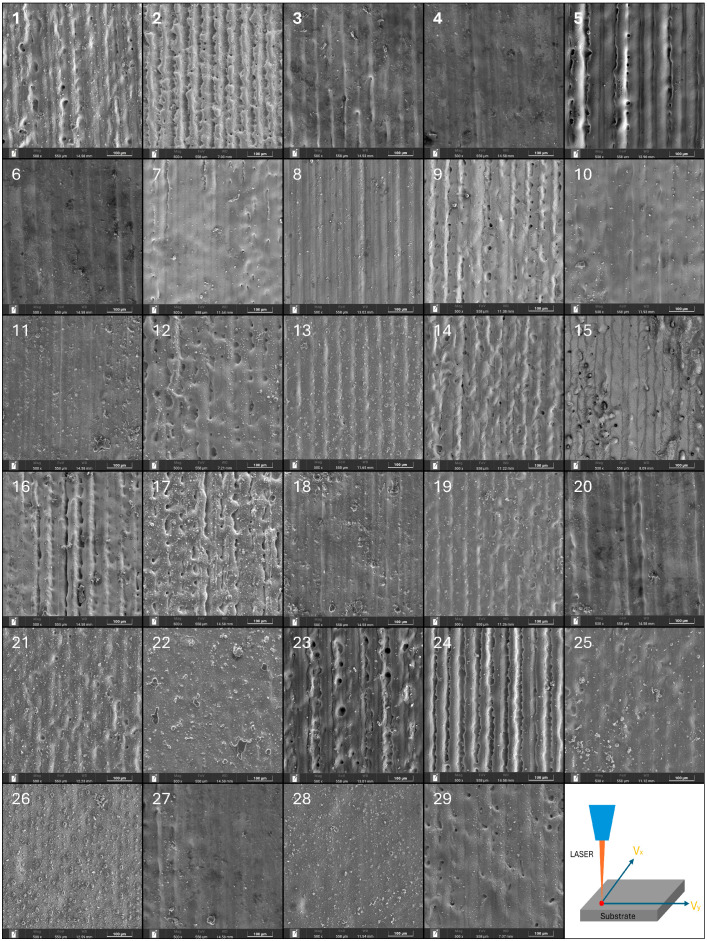
Surface topography of each sample after laser cleaning.

After the cleaning experiment, the surface removal rate (R%) was calculated using Image-Pro Plus 6 software. The surface morphology of the cleaned sample was observed using a field-emission scanning electron microscope (SEM, SU8020,Hitachi, Tokyo, Japan) and the oxygen content (O%) on the surface was analyzed with the attached energy-dispersive spectrometer (EDS, EX 350i, HORIBA, Paris, France). The maximum detection depth of the instrument is approximately 1 μm. Additionally, a 3D optical profilometer (Sensofar S neox 090, Sensofar, Barcelona, Spain) was employed to measure the surface roughness (Sa). The matrix and results of the experimental design are presented in Table 6, and the surface morphology images of the samples after cleaning corresponding to the experimental sequence in Table 6 are shown in Figure 5.

## 3. Effects of Parameters on Quality Characteristics

### 3.1. Mathematical Relationships and Analysis of Variance

The quadratic polynomials constructed from the response surface test describing the functional relationship between the input variables and the response values are as follows:

$$\left\{\begin{array}{l} \begin{array}{ll} f(1)= & 135.2185-1.693x_{1}-0.580x_{2}-0.0017x_{3}-0.6870x_{4}-0.004x_{1}x_{2}-1.7875\times 10^{-5}x_{1}x_{3}\\ & -0.0056x_{1}x_{4}-1.5\times 10^{-7}x_{2}x_{3}+0.0012x_{2}x_{4}+1.6875\times 10^{-6}x_{3}x_{4}+0.04105x_{1}^{2}\\ & +0.0015x_{2}^{2}+2.5902\times 10^{-7}x_{3}^{2}+0.0034x_{4}^{2}, \end{array}\\ \begin{array}{ll} f(2)= & 176.1046-1.8118x_{1}-0.3281x_{2}-0.0032x_{3}-0.1788x_{4}+0.0009x_{1}x_{2}+0.0001x_{1}x_{3}\\ & +2.5\times 10^{-5}x_{1}x_{4}+6.5\times 10^{-7}x_{2}x_{3}-0.0003x_{2}x_{4}-1.65\times 10^{-5}x_{3}x_{4}+0.0189x_{1}^{2}\\ & +0.0006x_{2}^{2}-7.114\times 10^{-8}x_{3}^{2}+0.0017x_{4}^{2} \end{array}\\ \begin{array}{ll} f(3)= & 8.8716+0.0099x_{1}+0.0028x_{2}-0.0005x_{3}-0.0795x_{4}+4.14\times 10^{-5}x_{1}x_{2}\\ & -5.75875\times 10^{-6}x_{1}x_{3}-0.0003x_{1}x_{4}-4.3875\times 10^{-7}x_{2}x_{3}-1.8175\times 10^{-5}x_{2}x_{4}\\ & +2.8563\times 10^{-6}x_{3}x_{4}+0.0013x_{1}^{2}-3.866\times 10^{-7}x_{2}^{2}+3.7352\times 10^{-8}x_{3}^{2}+0.00026x_{4}^{2}. \end{array} \end{array}\right.$$

Here, *f*(1) represents the surface oxygen content, *f*(2) represents the rust layer removal rate, *f*(3) represents the surface roughness, *x*_1_ represents the laser power, *x*_2_ represents the cleaning speed, *x*_3_ represents the scanning speed, and *x*_4_ represents the repetition frequency.

Table 7 presents the analysis of variance (ANOVA) results for the three response values. The *p*-values for the oxygen content, rust layer removal rate, and surface roughness model are all <0.0001, the degrees of freedom (F-values) are large, and the values of the misfit terms are all >0.1, indicating that the selected confidence interval is reasonable and the model is effective. Correlation coefficients were used in this study to verify the reliability of the fit; e.g., the correlation squared coefficient was R^2^ = 0.9723 for the oxygen content model, indicating that 97.23% of the experimental data can be explained by this model. Similarly, both the rust layer removal rate model and the surface roughness model were shown to be reliable through their correlation coefficients. The signal-to-noise ratios for all three response values were >4, indicating that the test results were reliable.

**Figure 6:**
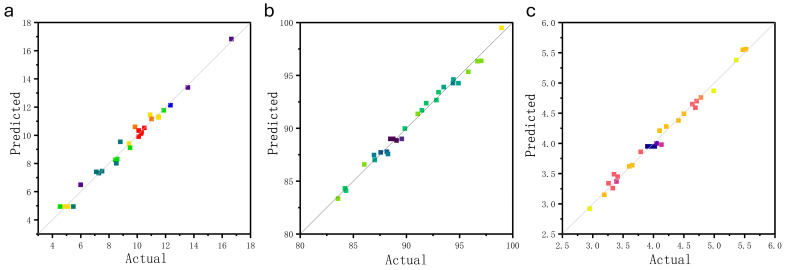
Comparison of actual and predicted values: (**a**) oxygen content; (**b**) removal rate; (**c**) roughness.

Residual plots corresponding to the oxygen content, rust layer removal rate, and surface roughness models are shown in Figure 6. All the points are distributed around a straight line. Normally distributed probabilities closer to the straight line indicate a better fit.

In summary, the developed laser cleaning agent model establishes a close-to-real nonlinear relationship between the two factors with good significance and high prediction accuracy.

### 3.2. Effects of Parameters of Laser Cleaning and Rust Removal Process on Surface Quality

#### 3.2.1. Effects of Process Parameters on Oxygen Content

**Figure 7:**
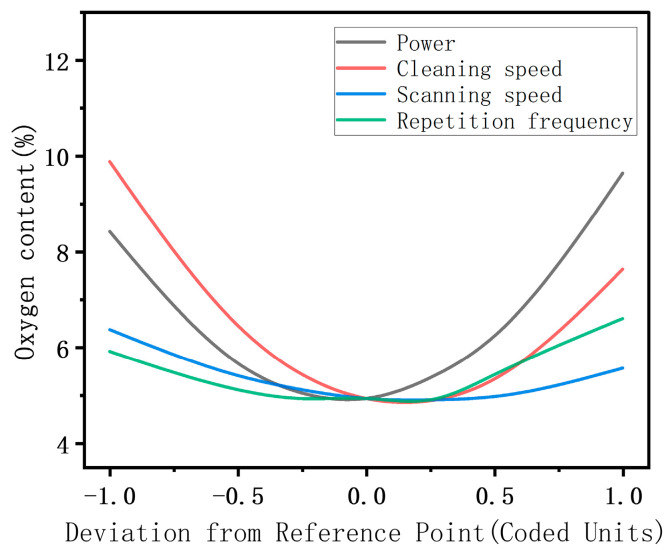
The impact of interactions among various factors on oxygen content.

According to the results from “Table 6: Response values of the ANOVA”, factors that significantly affect the oxygen content on the surface include the following: A, B, C, D, AB, AD, BD, A^2^, B^2^, C^2^, and D^2^. Among individual factors, the effects of cleaning speed and laser power are more significant than the other two parameters. The analysis shows that the interactions between power, cleaning speed, and repetition frequency, as well as between cleaning speed and scanning speed, are significant. Figure 7 demonstrates how these interactions influence the oxygen content. It is observed that in the lower-level range, the impact of laser power exceeds that of cleaning speed whereas in the higher level range, the influence of cleaning speed becomes more pronounced. However, merely changing the laser’s scanning speed and repetition frequency has no significant effect on the surface oxygen content.

**Figure 8:**
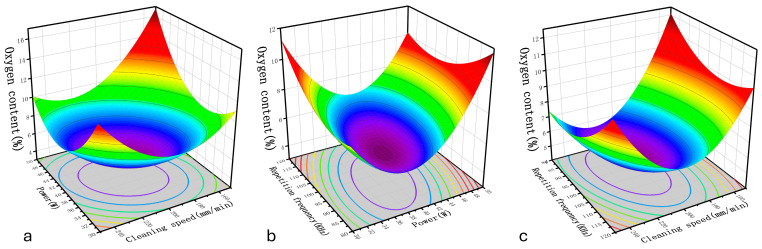
Effects of the cleaning process parameters on the oxygen content: (**a**) power vs. cleaning speed; (**b**) power vs. repetition frequency; (**c**) cleaning speed vs. repetition frequency.

As can be seen from Figure 8, the elemental oxygen content increases with an increase or decrease in the cleaning speed, which is explained as follows. Under excessive cleaning speeds, the temperature field in the thicker area of the surface rust layer does not fully reach the vaporization point of the rust layer, causing oxidized particles to remain on the substrate surface after cleaning [27]. When the cleaning speed is too low, there is a sharp increase in the surface temperature field, resulting in a prominent “ploughing effect” on the sample surface and ablation of the substrate, which causes the secondary oxidation of the substrate surface, increasing the surface oxygen content. The laser output energy can be effectively controlled by adjusting the laser output power and repetition frequency. In the circumstance of a higher cleaning speed, increasing the laser power can enhance the cleaning of residual oxidized particles, reducing the surface oxygen content, and when the cleaning speed is low, excessive power aggravates the substrate ablation. Increasing the laser repetition frequency reduces the laser energy density and increases the overlap rate of the laser spot. The two factors affect each other, weakening the effect of the laser repetition frequency on the temperature field. Therefore, the effect of the laser repetition frequency on the surface oxygen content is insignificant compared with those of the laser power and cleaning speed. As shown in Figure 8, the surface oxygen content can be reduced by using a combination of the median parameter values of the cleaning speed, laser power, and repetition frequency.

#### 3.2.2. Effects of Process Parameters on Removal Rate

**Figure 9:**
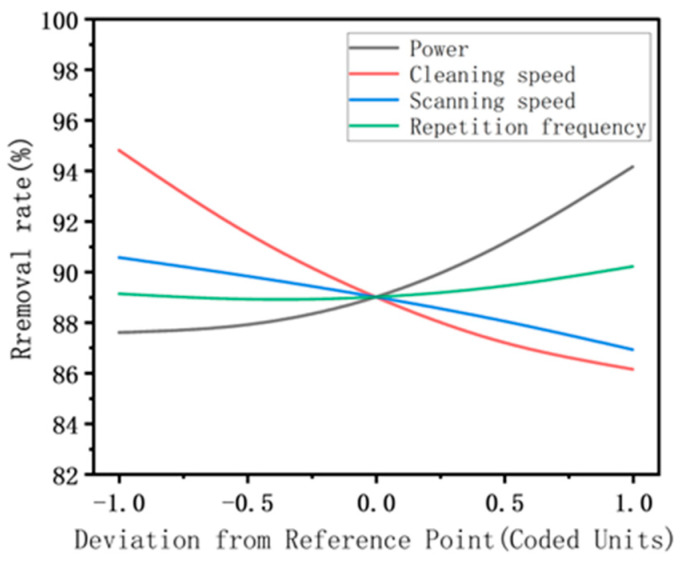
The impact of interactions among various factors on removal rate.

Combining the response values of the ANOVA from Table 6 with the effects of the interactions of various factors shown on the removal rate in Figure 9, it is evident that the significant factors influencing the removal rate of the rust layer are A, B, C, D, AC, CD, A^2^, B^2^, and D^2^, among which the single factors with the most significant influence are the laser cleaning speed and laser power, also the change law of laser power is opposite to that of cleaning speed.

**Figure 10:**
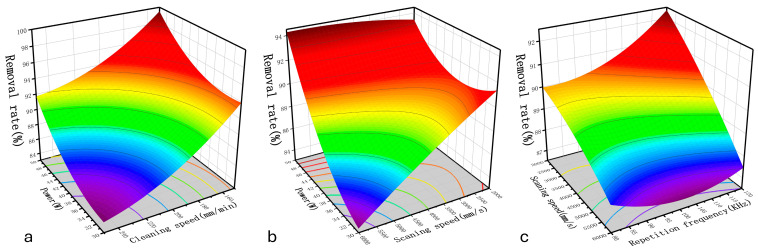
Effects of process parameters on the removal rate: (**a**) power vs. cleaning speed; (**b**) power vs. scanning speed; (**c**) scanning speed vs. repetition frequency.

Figure 10 shows two significant interaction terms of cleaning speed and laser power and their combined interactions with regard to removal rate. As shown in Figure 10a, when the laser power increases and the cleaning speed decreases, the rust layer removal rate increases rapidly, reaching a maximum of 98.95%. This is because when the laser cleaning speed is reduced, the laser residence time per unit area is increased, implying that the rust layer absorbs more energy per unit time and vaporizes more material on the surface layer. Meanwhile, the increase in laser power increases the energy density input to the sample surface and thus increases the removal rate of the rust layer. In contrast, in Figure 10b, increases in the laser scanning speed reduce the superposition rate of the spot, weakening the cumulative thermal effect of the surface temperature field and hindering the removal of the rust layer. Figure 10c shows the interaction between the laser repetition frequency and scanning speed. With the combination of a low scanning speed and high repetition frequency, a high removal rate can be obtained. Therefore, according to the three-dimensional response surface model, the combination of a low cleaning speed, high laser power, low scanning speed, and high repetition frequency results in a high rate of removal of the rust layer.

#### 3.2.3. Effects of Process Parameters on Roughness

**Figure 11:**
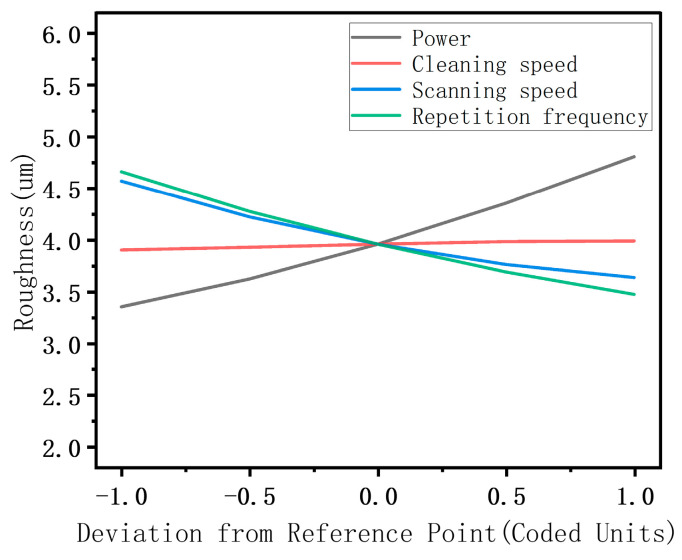
The impact of interactions among various factors on oxygen content surface roughness.

**Figure 12:**
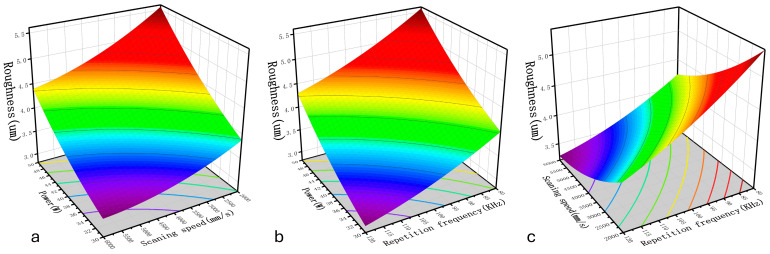
Effects of process parameters on the roughness: (**a**) power vs. scanning speed; (**b**) power vs. repetition frequency; (**c**) scanning speed vs. repetition frequency.

As shown in Table 7 and Figure 11, the factors that have a significant effect on the sample surface roughness after the cleaning process are A, C, D, AC, CD, A^2^, B^2^, and D^2^, among which the most significant single factor is the laser power. The laser power is an important parameter that determines the total energy of the laser output, and when it increases, the peak energy density of the laser increases, more energy is received at a single point, and the ablation becomes deeper, increasing the sample surface roughness. The factor with the second-most significant effect is the laser repetition frequency. Although increasing the laser repetition frequency reduces the spot overlap rate of the laser, it increases the output energy density of the laser. Therefore, as indicated by the interaction between the laser power and repetition frequency in Figure 12b, the surface roughness increases significantly with an increase in laser power and a reduction in repetition frequency and the interaction between the laser power and scanning speed follows a similar pattern. As shown in Figure 12a, simultaneously increasing the power and reducing the scanning speed strengthens the “ploughing effect” on the sample surface, which leads to deeper grooves on the substrate surface, significantly increasing the roughness of the substrate. Regarding the interaction between the laser repetition frequency and laser scanning speed, as shown in Figure 12c, when the repetition frequency increases, the effect of the scanning speed on the surface roughness weakens. In summary, for reducing the surface roughness, the laser power should be reduced and the scanning speed and repetition frequency should be increased.

### 3.3. Optimization of Surface Quality Characteristics after Cleaning Based on NSGA-II

The second-generation non-dominated sorting genetic algorithm (NSGA-II) is an algorithm for multi-objective optimization problems proposed by Deb et al. [28,29]. It ensures the diversity and excellence of solutions through rapid non-dominated ordering and crowding distance computation. In practice, NSGA-II combines binary tournament selection, crossover, and mutation to generate new populations and employs an elite strategy to maintain the propagation of superior solutions. It is suitable for finding the Pareto-optimal solution sets and has been widely used to solve complex multi-objective optimization problems [30,31,32]. NSGA-II has several significant advantages over particle swarm optimization [33,34] and other genetic algorithms [35]. First, it does not compute the algorithmic fitness function and weights; nor does it need to perform second-order fitting based on function fitting. Second, it is highly adaptable to complex problems, such as non-convex and nonlinear problems, and does not require excessive parameter tuning [36]. Thus, it has good performance for multi-objective optimization problems—especially in maintaining diversity and finding Pareto-optimal solutions.

In the previous study described in Section 3, a mathematical model was developed using Response Surface Methodology (RSM) to solve a multi-objective optimization problem. This problem involved process parameters such as laser power, cleaning speed, scanning speed, and repetition frequency, with the objective variables being oxygen content, removal rate, and surface roughness. The oxygen content and roughness needed to be minimized, while the removal rate needed to be maximized. Typically, optimization problems mean finding the minimum value of a function; therefore, when the minimization of an objective is required, the straightforward method is to solve for the minimum value. To address the removal rate function within a unified optimization framework, the problem of objective maximization is transformed into a minimization problem by prefixing the objective function of the removal rate with a negative sign, ensuring that all optimization objectives are efficiently dealt with. Thus, the following functional equation is obtained:

$$minF\left(x_{1},x_{2},x_{3},x_{4}\right)=\left\{\begin{array}{c} minF_{1}\left(x_{1},x_{2},x_{3},x_{4}\right)\\ maxF_{2}\left(x_{1},x_{2},x_{3},x_{4}\right)\\ minF_{3}\left(x_{1},x_{2},x_{3},x_{4}\right) \end{array}\;\;\;subject\;\;\;to\;\;\;\left\{\begin{array}{c} 30\leq x_{1}\leq 50\\ 150\leq x_{2}\leq 250\\ 2000\leq x_{3}\leq 4000\\ 80\leq x_{4}\leq 120 \end{array}\right.\right.$$

**Figure 13:**
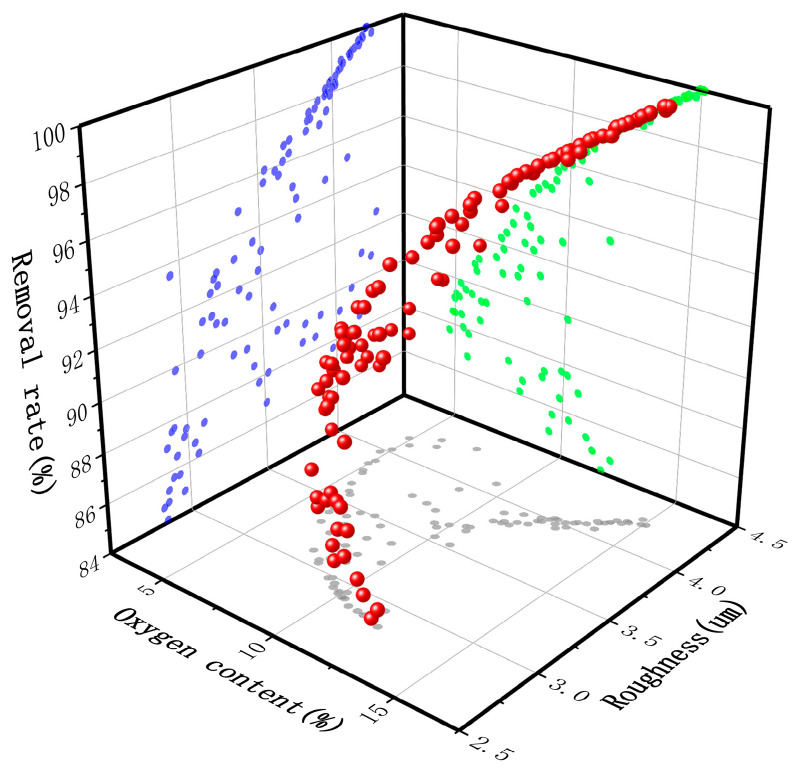
Pareto-optimal solution sets.

NSGA-II was used to obtain the Pareto-optimal solution sets, as shown in Figure 13. The parameters were set as follows: the population size was 250, the number of iterations was 200, and the crossover ratio was 0.85. The obtained Pareto-optimal solution set contained 105 groups of solutions. Results from the algorithm indicated that there are constraints among the optimal solution set and that the surface removal rate (*F*_3_) decreases when the surface oxygen content (*F*_1_) or surface roughness (*F*_2_) of the sample decreases.

On the basis of the actual laser cleaning process and steel surface treatment standards [37], an evaluation standard for the laser cleaning of Q390 steel rust layers is proposed. After laser cleaning, the substrate surface should exhibit a clear metal luster with no rust layer residue, the oxygen content should be controlled at ≤10 wt.%, the surface removal rate should be ≥90% [23], and the laser-cleaned sample surface should be smooth enough in cases where light spot cleaning marks are permitted to make sure the flowability of the weld pool reaches the standard during the subsequent welding process, to enhance the bonding between the weld metal and the base material, and to improve the stress corrosion cracking resistance of the material [38,39].

**Figure 14:**
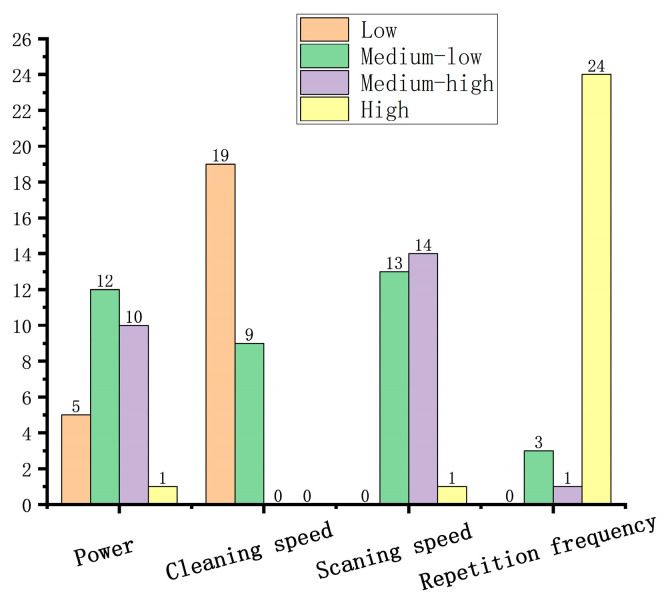
Number of intervals for each variable.

According to the aforementioned criteria, there are 28 groups of solutions that meet the requirements, and the corresponding number of intervals for each variable is shown in Figure 14. For most of the optimal solutions, the laser power is in the range of 35–45 W. Similarly, for the scanning speed, the optimal solutions are mainly concentrated in the center segment. When the cleaning speed is low, all the optimal solutions are within the interval of 150–200 mm/min. Meanwhile, most of the satisfactory repetition frequencies are concentrated in the interval of 110–120 kHz.

### 3.4. Optimization Results and Validation of Surface Quality and Laser Process Parameters

**Figure 15:**
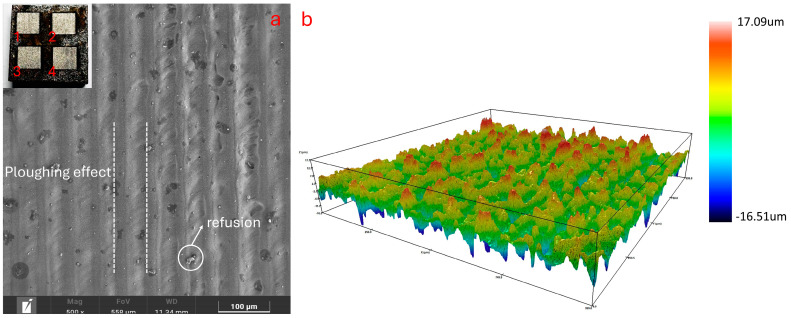
Verifying the results of the experiment: (**a**) SEM surface topography; (**b**) surface roughness.

According to the standard requirements of laser cleaning, four groups were randomly selected from the twenty-eight groups of satisfactory solutions for verification experiments, and the results are presented in Table 8. The error ranges of all the objective values were ≤5%, the surfaces of the samples exhibited a clear metallic luster, and the oxygen content was <10%. The No. 1 area was selected to examine its surface morphology, and the results are shown in Figure 15. The surface rust layer was completely removed and the surface roughness of the sample was appropriately reduced while allowing cleaning marks to remain. According to the laser cleaning evaluation method mentioned above, the rust removal effect was good and the rust removal efficiency was high. The resulting optimal parameter set was as follows: a laser power of 44.99 W, cleaning speed of 174.01 mm/min, scanning speed of 3852.03 mm/s, and repetition frequency of 116 kHz. By comparing its results with the multi-objective optimization results generated using the Design Expert software (refer to Table A1 in Appendix A), it was found that its accuracy is significantly higher than the accuracy of the optimization results obtained through the response surface method.

## 4. Conclusions

The objective of this study was to explore the optimization strategy of the laser cleaning process for removing the surface rust layer of Q390 steel through experimental methods. RSM was applied to design the experiments and build the corresponding statistical models, and the models were optimized using NSGA-II to accurately predict the surface quality after laser cleaning. This paper has detailed the experimental design, model construction, and optimization process. The accuracy of the model predictions was verified through experiments. The results of the experimental analysis were as follows:

(1) The relationship between the objective value and the input variables had been effectively represented by the proxy mode based on the response surface, and evaluation parameters such as the *p*-value, F-value, and signal-to-noise ratio indicated that the proxy model was well fitted.
(2) The pattern of the influence of each single factor on the surface cleaning quality after secondary laser cleaning was analyzed, along with the influence pattern for the interaction of laser power, cleaning speed, scanning speed, and repetition frequency. For the surface oxygen content, the single factors with the most significant influence were the cleaning speed and laser power, and the median value of each process parameter could be selected to minimize the oxygen content. Regarding the surface removal rate, the combination of a low cleaning speed, high laser power, and low scanning speed resulted in the highest removal rate. The single factor most significantly affecting the surface roughness was the laser power, and the surface roughness could be reduced by increasing the scanning speed and repetition frequency.
(3) On the basis of the mathematical model constructed using the response surface, NSGA-II was employed to find the optimum, from which good objective values were obtained, and the optimal process parameters were obtained as follows: a laser power of 44.99 W, cleaning speed of 174.01 mm/min, scanning speed of 3852.03 mm/s, and repetition frequency of 116 kHz.

In this paper, a new optimization method for laser cleaning parameters of carbon steel rust layers has been proposed, and the influence of parameters on the target value has been studied, which has provided effective support for practical production applications. It is hoped that a comprehensive reference framework for laser cleaning process parameters of carbon steel will be constructed on this basis to guide and improve the cleaning process in industrial applications. Next, attempts will be made to further optimize the NSGA-II algorithm and to integrate it with the BP genetic algorithm in order to analyze the optimization effects of laser cleaning process parameters.

## Figures and Tables

**Table 1:** The types of lasers used by other researchers.

Substrate	Laser Type	Ref.
Q345	Solid-state laser with flat-top laser energy	[10]
AH36	Ultraviolet nanosecond laser	[11]
Q345	Nanosecond pulsed laser	[3]
Mild steel	Nanosecond pulsed laser	[12]
Q235B	1080 nm continuous laser	[13]

**Table 2:** Chemical composition of Q390 steel.

Element	Fe	C	Mn	Si	S, P	Al	C_eq_
Content (wt.%)	base	≤0.20	1.00–1.60	0.55	≤0.030	0.038	0.37–0.42

**Table 3:** Main thermophysical parameters of carbon steel plate substrate and rust layer [13,19].

Thermophysical Parameter	Carbon Steel	Rust Layer
Destiny/(kg·m^−3^)	7860	5200
Thermal conductivity/(W·m^−1^·K^−1^)	44.5	4.3
Constant pressure heat capacity/(J·kg^−1^·K^−1^)	600	900
Absorption ratio	0.49	0.8
Melting temperature/K	1808	1773
Vaporization temperature/K	3133	2973

**Table 4:** Ranges of the main parameters of the laser cleaning platform.

Parameter	Value
Wavelength	1064 nm
Pulse width	100 ns
Focused spot diameter	100 µm
Laser power	0–100 W
Repetition frequency	10–100 kHz
Scanning speed	0–8000 mm/s
Cleaning speed	0–1000 mm/min

**Table 5:** BBD experiment factor and level design.

Variable	Code		
	Low (−1)	Medium (0)	High (1)
Laser power (W)	30	40	50
Laser cleaning speed V_y_ (mm/min)	150	200	250
Laser scanning speed V_x_ (mm/s)	2000	4000	6000
Laser repetition rate f (kHz)	80	100	120

**Table 6:** Experimental design matrix and experimental results.

Run	P	V_y_	V_x_	f	O%	R%	Sa
1	40	300	4000	80	7.11	86.04	4.7125
2	50	200	4000	80	11.89	94.89	5.5212
3	40	200	4000	100	5.49	88.49	3.8995
4	40	200	4000	100	4.57	88.64	3.9142
5	50	300	4000	100	10.28	91.45	4.9915
6	40	200	4000	100	4.88	88.75	3.9867
7	40	200	6000	80	6.01	87.58	4.0968
8	30	200	2000	100	8.81	91.47	3.7948
9	50	200	6000	100	10.12	94.38	4.4054
10	30	200	4000	120	11.53	89.06	2.9451
11	40	300	6000	100	8.45	84.31	3.6047
12	50	100	4000	100	16.67	98.95	4.7757
13	30	200	6000	100	9.43	83.56	3.1897
14	40	100	2000	100	11.52	97.03	4.5034
15	50	200	4000	120	10.32	95.82	4.2125
16	40	200	2000	80	7.52	89.85	5.3625
17	40	100	4000	80	12.36	94.42	4.6921
18	40	200	2000	120	8.53	91.86	4.1341
19	40	200	4000	100	4.55	89.56	4.0245
20	40	200	4000	100	5.12	89.56	3.9456
21	30	200	4000	80	8.59	88.15	4.0525
22	40	200	6000	120	7.29	86.95	3.3254
23	40	300	2000	100	9.51	88.26	4.6378
24	50	200	2000	100	10.93	96.02	5.4712
25	40	300	4000	120	9.86	87.02	3.3534
26	30	300	4000	100	13.59	84.21	3.3895
27	40	100	4000	120	10.12	96.69	3.4057
28	30	100	4000	100	11.03	93.57	3.2565
29	40	100	6000	100	10.52	92.82	3.6458

**Table 7:** Response values of the ANOVA.

Variance Source	Response Value					
	Oxygen Content		Removal Rate		Roughness	
	F-Value	*p*-Value	F-Value	*p*-Value	F-Value	*p*-Value
Model	71.16	<0.0001	96.5	<0.0001	105.78	<0.0001
A	19.09	0.0006	376.23	<0.0001	695.67	<0.0001
B	65.76	<0.0001	673.93	<0.0001	1.53	0.2366
C	9.13	0.0092	114.53	<0.0001	288.66	<0.0001
D	6.35	0.0245	10.38	0.0061	453.14	<0.0001
AB	87.74	<0.0001	2.41	0.1428	0.1869	0.6721
AC	2.24	0.1567	58.53	<0.0001	5.79	0.0305
AD	22.28	0.0003	0.0003	0.9865	1.1	0.311
BC	0.0039	0.9508	0.0503	0.8258	0.8397	0.375
BD	27.28	0.0001	1.24	0.2846	0.1441	0.7099
CD	0.0799	0.7816	5.19	0.039	5.69	0.0317
A^2^	478.89	<0.0001	68.8	<0.0001	11.71	0.0041
B^2^	417.69	<0.0001	41.59	<0.0001	0.0007	0.9799
C^2^	30.51	<0.0001	1.56	0.2317	15.79	0.0014
D^2^	51.59	<0.0001	8.68	0.0106	7.75	0.0146
Lack of fit	1.64	0.3345	1.34	0.4167	4.42	0.0826
R^2^	0.9861		0.9897		0.9906	
Adjusted R^2^	0.9723		0.9795		0.9813	
Predicted R^2^	0.9316		0.9508		0.9493	
Adeq precision	34.6257		38.7301		38.2623	

**Table 8:** Optimization results and experimental validation.

No	P/W	V_y_/(mm/min)	V_x_ (mm/s)	f/kHz	O%		E%	R%		E%	Sa/µm		E%
					Pre	Act		Pre	Act		Pre	Act	
1	44.59	174.01	3852.03	116	8.65	8.48	2.01%	94.71	93.85	0.9%	3.9415	3.9039	0.96%
2	33.31	173.29	4691.38	114	8.29	8.41	1.42%	90.05	90.82	0.85%	3.0111	3.1542	4.53%
3	41.42	155.18	3922.68	117	9.71	9.59	1.25%	95.85	93.11	2.94%	3.6094	3.6854	2.06%
4	44.29	177.49	3707.94	99	7.31	7.13	2.52%	92.57	93.77	1.27%	4.2975	4.3512	1.23%

## Data Availability

The original contributions presented in the study are included in the article, further inquiries can be directed to the corresponding author.

## Appendix A

### Appendix A.1. Computational Formul

Laser fluence [23,40]:

$$F_{0}=\frac{4P}{\pi d^{2}f}$$

Laser energy density refers to the energy distributed by a laser beam over a unit area. It is calculated by dividing the total energy of the laser beam by the area it irradiates.

Laser spot overlap rate [23,40]:

$$\eta =\left(1-\frac{v}{f\cdot d}\right)\times 100\%$$

In laser processing, the overlap ratio of laser spots refers to the degree of mutual overlap of successive laser spots on the surface of the workpiece.

In the formula, *P* represents the average laser power; *d* denotes the focused diameter of the laser spot (mm), *f* indicates the pulse repetition frequency (Hz), and *v* represents the scanning speed (mm/s).

### Appendix A.2. Response Surface Multi-Objective Optimization Results

Parameter settings were conducted in Design Expert software, setting the weights for the oxidation amount and removal rate at 5, and the weight for roughness at 4, for optimization. Three sets of parameters were randomly selected from the results for experimental verification. The parameter sets and verification results are shown in Table A1 below.

**Table A1:** Multi-objective solution set based on Design Expert.

No	P/W	V_y_/(mm/min)	V_x_ (mm/s)	f/kHz	O%		E%	R%		E%	Sa/µm		E%
					Pre	Act		Pre	Act		Pre	Act	
1	37.168	162.73	3354.06	120	8.841	9.294	4.8%	94.81	90.12	5.23%	3.390	3.7812	10.34%
2	44.845	183.442	4834.93	120	8.027	9.812	18.1%	93.30	91.56	1.90%	3.681	3.8754	5.01%
3	37.232	163.63	3311.70	119	8.766	8.156	7.47%	94.75	89.22	6.19%	3.402	3.7185	8.51%
